# SUPPORTING FAMILY CAREGIVING IN MULTICULTURAL CONTEXTS

**DOI:** 10.1093/geroni/igae098.0695

**Published:** 2024-12-31

**Authors:** Weichao Yuwen, Maggie Ramirez, Serena Jinchen Xie

**Affiliations:** Chair; Co-Chair; Discussant

## Abstract

There are 53 million family caregivers providing an estimated $470 billion in unpaid care services in the U.S. annually. Over 40% of them self-identify as having a racial or ethnic minority background, and at least one-third speak a language other than English at home. Caregivers from racial and ethnic minority groups experience significantly more physical and emotional stress than their white counterparts. This session focuses on supporting family caregiving in multicultural contexts. Berridge will discuss cultural values as they relate to technology intervention considerations for caregivers of older adults. Li and Liang will discuss a study exploring the needs of Chinese American families caring for older adults and their perceptions of using technologies such as chatbots. Specifically, Li will discuss results from four focus groups with community-based organizations’ staff supporting these families, and Liang will discuss results from family caregivers. Cardoso-Arias will discuss structural barriers to accessing supportive services among Latino family caregivers. Finally, Aguilar will describe a conceptual model of family caregiving and health equity. To conclude, our discussant Ms. Xie will discuss cross-cutting implications across studies and offer insights on how to incorporate cultural considerations in developing technologies to support family caregivers.

